# Quantification of Process Induced Disorder in Milled Samples Using Different Analytical Techniques

**DOI:** 10.3390/pharmaceutics2010030

**Published:** 2010-02-16

**Authors:** Ulrike Zimper, Jaakko Aaltonen, Cushla M. McGoverin, Keith C. Gordon, Karen Krauel-Goellner, Thomas Rades

**Affiliations:** 1 School of Pharmacy, University of Otago, 18 Frederick Street, 9016 Dunedin, New Zealand; E-Mail: ulrike.zimper@otago.ac.nz (U.Z.); 2 School of Pharmacy, Faculty of Health Sciences, University of Eastern Finland, Yliopistonranta 1 C, 70210 Kuopio, Finland; E-Mail: jaakko.aaltonen@uef.fi (J.A.); 3 Department of Chemistry, MacDiarmid Institute for Advanced Materials and Nanotechnology, University of Otago, Union Place West, 9016 Dunedin, New Zealand; E-Mails: cmcgoverin@chemistry.otago.ac.nz (C.M.M.); Kgordon@chemistry.otago.ac.nz (K.C.G.); 4 Institute of Food, Nutrition and Human Health, Massey University, 63 Wallace Street, 6021 Wellington, New Zealand; E-Mail: K.Krauel-Goellner@massey.ac.nz (K.K.-G.)

**Keywords:** indomethacin, simvastatin, amorphous, process induced disorder, ball milling, DSC, XRPD, Raman spectroscopy, multivariate analysis, principal component analysis (PCA), partial least squares regression (PLS)

## Abstract

The aim of this study was to compare three different analytical methods to detect and quantify the amount of crystalline disorder/ amorphousness in two milled model drugs. X-ray powder diffraction (XRPD), differential scanning calorimetry (DSC) and Raman spectroscopy were used as analytical methods and indomethacin and simvastatin were chosen as the model compounds. These compounds partly converted from crystalline to disordered forms by milling. Partial least squares regression (PLS) was used to create calibration models for the XRPD and Raman data, which were subsequently used to quantify the milling-induced crystalline disorder/ amorphousness under different process conditions. In the DSC measurements the change in heat capacity at the glass transition was used for quantification. Differently prepared amorphous indomethacin standards (prepared by either melt quench cooling or cryo milling) were compared by principal component analysis (PCA) to account for the fact that the choice of standard ultimately influences the quantification outcome. Finally, the calibration models were built using binary mixtures of crystalline and quench cooled amorphous drug materials. The results imply that the outcome with respect to crystalline disorder for milled drugs depends on the analytical method used and the calibration standard chosen as well as on the drug itself. From the data presented here, it appears that XRPD tends to give a higher percentage of crystalline disorder than Raman spectroscopy and DSC for the same samples. For the samples milled under the harshest milling conditions applied (60 min, sixty 4 mm balls, 25 Hz) a crystalline disorder / amorphous content of 44.0% (XRPD), 10.8% (Raman spectroscopy) and 17.8% (DSC) were detected for indomethacin. For simvastatin 18.3% (XRPD), 15.5% (Raman spectroscopy) and 0% (DSC, no glass transition) crystalline disorder/ amorphousness were detected.

## 1. Introduction

Milling is a common unit operation in the pharmaceutical industry to diminish the particle size of powders and/ or to achieve narrower size distributions for better mixing or for other reasons, such as the use of powders in pulmonary drug delivery and for dissolution rate improvement of poorly water soluble drugs [1]. New active pharmaceutical ingredients (API) are often very poorly water soluble, and improvement of the dissolution rate of such APIs is one of the most important topics of research in the field of pharmaceutics and dosage form development. According to the Noyes-Whitney equation the dissolution rate is directly proportional to the surface area and solubility of a particle [2]. Reducing the particle size is a simple and commercially and technically feasible approach to improve the dissolution rate by increasing the overall surface area of a powder [3]. However, particle size is often not the only property of a solid particle that is affected by milling. During the milling process the materials are put under mechanical stress that can cause solid state transformations including polymorphic conversion, introduction of crystalline disorder or the formation of amorphous domains [4,5,6]. Since different solid state forms of a given API often have different physical properties such as differences in flowability, hygroscopicity and chemical and physical stability but also different solubilities and dissolution rates (and hence possibly different bioavailability), it is necessary to monitor solid state changes during a milling operation. 

There are many analytical tools that may be used for the quantification of crystalline disorder or amorphous content of APIs [7]. Among these, X-ray powder diffraction (XRPD), differential scanning calorimetry (DSC) and Raman spectroscopy are all established analytical methods, but they probe the solid state at different levels (particulate *versus* molecular level) [8]. Therefore the information gained by these methods may be used in a complementary way. 

XRPD is a diffraction technique that probes lattice properties of crystalline solids. The resulting XRPD diffractograms show the presence or lack of crystal planes in the sample. As XRPD essentially measures a crystalline property, a reduction in the peak heights and areas and the appearance of a “halo” in the diffractogram is linked to a reduction in crystallinity, which may then be interpreted as increase in crystalline disorder or amorphousness. However, particle size reduction during milling may also cause a similar change in the diffractogram (*i.e.*, a reduction in peak heights and peak areas) [9].

DSC is a thermal method that measures heat capacity changes within the sample and therefore is capable of detecting phase transitions such as the glass transition of an amorphous drug. In contrast to XRPD measurements the change in heat capacity (∆c_p_) at the glass transition temperature (T_g_) can be used as direct measurement of amorphous material rather than as a sum of truly amorphous and otherwise disordered material

Raman spectroscopy, as a vibrational spectroscopic method, probes properties of the molecule itself, and changes in the solid state properties of an API are inferred from changes in the molecular conformation and molecular environment due to different packing conditions of the molecules in the different solid forms. These changes become apparent as subtle changes in the peak positions and intensities in the Raman spectra. 

All three methods mentioned above can be used for qualitative as well as quantitative solid state analysis. The response of each of these analytical techniques to increased crystalline disorder induced by milling and the complementarities and agreement between DSC, XRPD and Raman spectroscopy in the quantification of disorder within milled indomethacin and simvastatin was investigated in this study. Analysis of whole spectra and diffractograms can be facilitated by multivariate analytical methods such as principal component analysis (PCA) and partial least squares regression (PLS) analysis [10]. Therefore, these tools have been applied in the current study.

Melting followed by quench cooling is regularly applied to convert a crystalline drug into an amorphous form [11,12,13]. However, milling (especially under cryogenic conditions) as preparation method to achieve fully amorphous compounds also appears possible and has been performed in several studies [5,14,15,16,17]. This process though requires longer milling times and different process parameters (e.g., number and size of milling balls used in a ball milling operation) than applied in milling processes where the objective is to reduce the particle size. Therefore, melt quench cooled amorphous and cryo milled amorphous indomethacin have been compared with regard to their suitability as an amorphous standard in this study.

The aim of this work was to address the problem that different results with respect to reduced crystallinity (increased crystalline disorder and/ or amorphousness) of milled APIs may be detected when using different analytical methods. Besides fully crystalline material and completely amorphous material, different forms of the solids with increasing disorder may be formed during a milling operation. The problem thus arises that different techniques may detect these disordered solid forms differently, thus potentially leading to different results in the determination of the degree of crystallinity of a given sample.

Furthermore, the problem of finding a suitable calibration standard for an amorphous/ crystalline mixture is discussed. It is an inherent problem that independently of the preparation method chosen for the amorphous standard, the resulting calibration standard may only be an approximation of the actual induced crystalline disorder in the milling process. 

In summary, for the quantification of crystalline disorder in milled samples three aspects have been considered: analytical method and the calibration standard used and the drug itself.

## 2. Experimental Section

### 2.1. Materials

**Figure 1:**
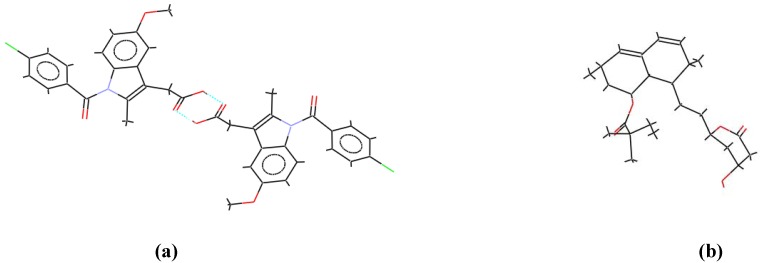
Molecular structures of **(a)** γ indomethacin dimer and **(b)** simvastatin obtained from the Cambridge Structural Database (CSD) and Mercury 2.2 software (refcode INDMET for indomethacin and EJEQAL for simvastatin).

Indomethacin (γ form, M = 357.5 g/mol) (Hawkins Inc. USA, Lot PH05113013) and simvastatin (M = 418.6 g/mol) (Salutas Pharma, Germany, Batch No. B-710543/00157) were used as received (Figure 1). Both compounds are poorly water soluble and readily convert into the amorphous form upon milling [12,18]

### 2.2. Preparative Methods

#### 2.2.1. Sample preparation by ball milling (cold room)

The drugs were milled using an oscillatory ball mill (Mixer Mill MM301, Retsch GmbH & Co., Germany). A central composite face centered design was used for the milling experiments for both compounds. The experimental design was created using MODDE software (version 7, Umetrics AB, Sweden). A total of 600 mg powder was used for each milling operation. The sample powder were placed in 25 mL stainless steel jars and milled at milling frequencies of 5-25 Hz for 5-60 min with 3-60 stainless steel balls with a diameter of 4 mm (Table 1). Milling was conducted in a cold room at 4 °C. The processed samples were stored in air tight containers over silica gel at 4 °C and measured by DSC, XRPD and Raman spectroscopy within 24 hours after preparation.

#### 2.2.2. Cryo milling to produce an amorphous standard

**Figure 2:**
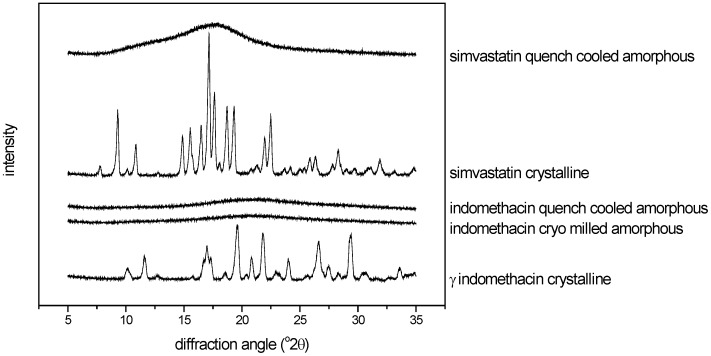
XRPD diffractograms of the crystalline, cryo milled amorphous and quench cooled amorphous compounds used for calibration (scale arbitrary).

An oscillatory ball mill (Mixer Mill MM301, Retsch GmbH & Co., Germany) was used for cryo milling as follows: 1 g of indomethacin was placed into a 25 mL milling jar and milled with two 12 mm balls for 120 minutes. The jars were immersed in liquid nitrogen for 3 min before milling and repeatedly for 3 min at 15 min intervals. Conversion to the amorphous state after preparation was confirmed by XRPD (Figure 2).

#### 2.2.3. Melt quench cooling to produce an amorphous standard

For melt quench cooling, the compounds were placed on a stainless steel plate and melted in an oven at 163 °C (indomethacin) and 142 °C (simvastatin). As soon as a clear melt was obtained, liquid nitrogen was poured onto the plate in order to solidify the melt. Subsequently, the melt quenched compounds were crushed and sieved through a 250 μm mesh. Conversion to the amorphous state after preparation was confirmed by XRPD (Figure 2).

#### 2.2.4. Preparation of binary mixtures

Amorphous forms for calibration were prepared by melt quench cooling for indomethacin and simvastatin and cryo-milling for indomethacin only, as described above [5,11]. Subsequently, the prepared amorphous forms of the drugs were geometrically mixed with their crystalline counterparts with a spatula in a glass vial. Mixing ratios ranged from 1:9 to 9:1 (amorphous: crystalline, w/w). Each mixing ratio was prepared in triplicate.

### 2.3. Analytical methods

#### 2.3.1. X-ray powder diffraction (XRPD)

XRPD analysis was performed using an X’Pert PRO X-ray diffractometer (PANalytical, The Netherlands; MPD PW3040/60 XRD; CuKα anode; λ = 1.541 Å). The samples were consolidated in an aluminium sample holder and scanned at 40 kV and 30 mA from 5 to 35 °2θ using a scanning speed of 0.1285 °2θ /min and a step size of 0.0084 °2θ. The diffractograms were collected using X’Pert High Score software (version 2.2.0) and plotted using OriginPro 7.0 (OriginLab Corporation, USA).

#### 2.3.2. Differential scanning calorimetry (DSC)

DSC thermograms (DSC Q100 V8.2 Build 268, TA Instruments, USA) were obtained under a nitrogen gas flow of 50 mL/min. Calibration of the DSC instrument was carried out using indium (for temperature and enthalpy) and sapphire (for heat capacity) as standards. Sample powders (approximately 3 mg, accurately weighed) were crimped in aluminium pans with sealed lids and heated at a scanning rate of 20 K/min from -10 to 200 °C. The heat capacity change (∆c_p_) at the glass transition temperature T_g_ (midpoint) was determined using TA Universal Analysis 2000 software (TA Instruments, USA). 

#### 2.3.3. FT-Raman-spectroscopy

FT-Raman spectra were obtained using a Bruker IFS 55 FT-Raman interferometer fitted with a Bruker FRA 106 S FT-Raman accessory (Bruker Optik GmbH, Germany). The instrument used a D418-T Ge diode detector and a Coherent Compass 1064-500N laser (Coherent Inc., USA). Analysis was carried out at room temperature with the wavelength of the Nd:YAG laser set at 1064 nm and the laser power set at 120 mW. Samples were packed in glass sample holders and spectra were collected at a resolution of 4 cm^-1^. The number of scans obtained per spectrum was 32. Sulphur was used as reference standard to monitor wave number accuracy. Data were collected using OPUS^TM^-software (Bruker Optik GmbH, Germany).

#### 2.3.4. HPLC

The HPLC system used consisted of a Shimadzu LC-10ATvp pump and a Shimadzu SPD-10Avp UV-vis detector (Shimadzu Corp., Kyoto, Japan) for both compounds. The analysis for indomethacin was performed following the method reported by Hess *et al*. [19] using a Phenosphere-NEXT 5μ C18 column (150mm x 4.6mm; Phenomenex, USA). The mobile phase consisted of 75% methanol and 25% of 0.2% phosphoric acid and was used at a flow rate of 1 mL/min. The injection volume was 100 μL. The UV-vis detector was set at 320 nm. The peak area correlated linearly with concentration in the range of 0.0125-0.25 mg/mL (r^2^ = 0.997), hence measurements were undertaken for drug concentrations of 0.025 mg/mL. The retention time of indomethacin was 7.1 min. For simvastatin the mobile phase consisted of 65 v/v% Acetonitrile/ 35 v/v% Milli-Q-water. The flow rate was 1 mL/min. The method applied was adapted from Meng *et al*. [20]. The UV-vis detector was set at 238 nm. The retention time for simvastatin was 12.2 min. Linearity of the standard curve was given in the range of 0.0012 mg/mL-0.024 mg/mL (r^2^ = 0.997), hence measurements were undertaken for drug concentrations of 0.012 mg/mL. All measurements were performed in triplicate.

#### 2.3.5. Multivariate Analysis

Principal component analysis (PCA) and partial least squares regression (PLS) (SIMCA-P 11 software, Umetrics AB, Sweden) were used to analyse the XRPD diffractograms and the Raman spectra. Whole diffractograms (ranging from 5 to 35 ^o^2θ) and spectra (ranging from 500 to 3500 cm^-1^) were used for the analysis. Cross validation was used to determine the optimal number of principal components (PCs) as well as PLS factors for the models, with Q^2^ (goodness of fit) as the criterion for the number of components/ factors used and R^2^ being the correlation coefficient. For the PLS models, cross validation was also used for the validation of the prediction and the root mean square error of estimation (RMSEE) was used as the criterion for the model performance. For Raman spectroscopic data, standard normal variate transformation (SNV) was applied as a preprocessing method and the spectra were mean centered prior to PCA and PLS modeling. XRPD data were neither preprocessed nor scaled prior to PCA modeling. PLS models for the XRPD data were computed using orthogonal signal correction (OSC) and mean centering as preprocessing and scaling methods.

#### 2.3.6. Statistical Analysis

One way analysis of variance (ANOVA) was performed with Minitab software (Minitab, State College PA, USA).

#### 2.3.7. Cambridge Structural Database (CSD)

Molecular structures were obtained from the CSD [21] using Mercury 2.2 software (refcodes INDMET and EJEQUAL, respectively).

## 3. Results and Discussion

### 3.1. Chemical stability

Using HPLC, it could be shown for both compounds that chemical degradation even under the harshest milling conditions applied (600 mg of compound milled for 60 min with sixty 4 mm balls at 25 Hz) was less than 3% (w/w). Previous studies also showed that chemical degradation was negligible for these compounds when subjected to quench cooling and cryo milling [5,11]. Therefore, it could be concluded that chemical degradation did not significantly affect the results obtained using the different analytical techniques and that the results obtained are due to reduction in crystallinity (milling induced crystalline disorder and/ or true amorphousness) and particle size reduction.

### 3.2. Influence of the calibration standard on the quantification outcome for process induced disorder

As shown in other studies, multivariate analysis is a powerful tool that can be used in form of PCA to characterize the solid state qualitatively [5,22]. In this study, PCA has been utilized in order to find a suitable calibration standard for the amorphous compounds. For this purpose, melt quench cooled amorphous/ crystalline and cryo milled amorphous/ crystalline binary mixtures of indomethacin were compared.

#### 3.2.1. PCA for Raman spectra of the binary mixtures of indomethacin

The Raman spectra of the two differently prepared binary mixture sets and the unprocessed (crystalline) and milled samples were subjected to PCA.

**Figure 3:**
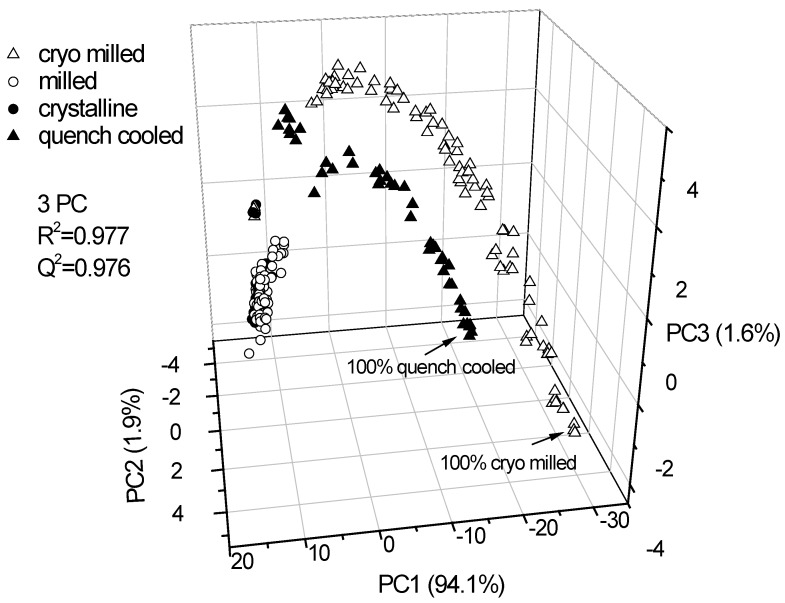
PCA scores plot of the Raman spectroscopic data of pure (unprocessed crystalline and ball milled) indomethacin forms and binary crystalline/ amorphous mixtures at different ratios. Displayed are the scores of cold room milled samples and unprocessed (crystalline) samples (hidden within the scores of the cold room milled samples) and quench cooled amorphous/ crystalline binary mixtures and cryo milled amorphous/crystalline binary mixtures. The PCs explain 94.1%, 1.9% and 1.6% of the spectral variance, respectively.

Three PCs explained 97.6% of the variance in the data. The score plot showed that Raman spectroscopy combined with PCA differentiates between cryo milled amorphous/ crystalline and quench cooled amorphous/ crystalline binary mixtures as well as the crystalline and milled samples (Figure 3). The milled samples cluster around the unprocessed crystalline samples and show a similar distance to either of the binary mixtures (cryo milled amorphous/ crystalline and quench cooled amorphous/ crystalline).

#### 3.2.2. PCA for XRPD data of the binary mixtures of indomethacin

**Figure 4:**
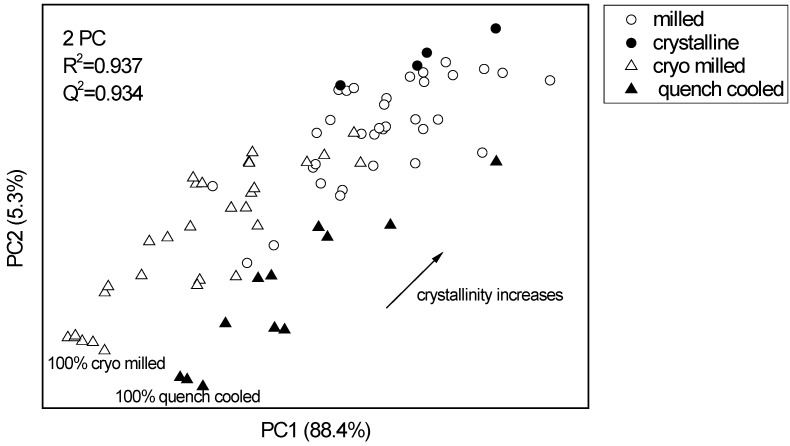
PCA scores plot of the XRPD data of pure indomethacin forms and binary crystalline/ amorphous mixtures at different ratios. The pure indomethacin samples (unprocessed crystalline and ball milled) are located between the binary cryo milled amorphous/ crystalline and quench cooled amorphous/ crystalline mixtures. The PCs explain 88.4% and 5.3% of the diffractive variance, respectively.

The diffractograms of the same indomethacin samples were also analysed using PCA, however, there was no clear distinction between cryo-milled/ crystalline and quench cooled/ crystalline binary mixtures. In the PCA scores plot the milled samples are placed in between the binary mixtures and again show a similar distance to either of the binary mixtures. Two PCs explained 93.7% of the variance in the data (Figure 4). No form α indomethacin could be detected by XRPD during the course of the experiments.

Overall, PCA revealed that neither of the binary mixture types (prepared using either melt quench cooled amorphous or cryo milled amorphous indomethacin) mimicked the process induced crystalline disorder of the cold room milling more than the other. Although cold room and cryo milling are both milling processes, they differ significantly as cold room milling is likely to favor recrystallisation, whereas cryo milling favors amorphisation [4]. Therefore melt quench cooling was chosen as preparative method to prepare an amorphous standard for the calibration as it has been shown earlier to be more stable than the cryo milled amorphous form [11,12]. Since the stability of the milled samples has to be taken into consideration as well, storing times after sample preparation have been kept to a minimum and all samples were measured by all three methods within 24 hours after preparation, starting with DSC and followed by XRPD and Raman spectroscopy.

#### 3.2.3. Thermal analysis of quench cooled amorphous and cryo milled amorphous indomethacin

**Figure 5:**
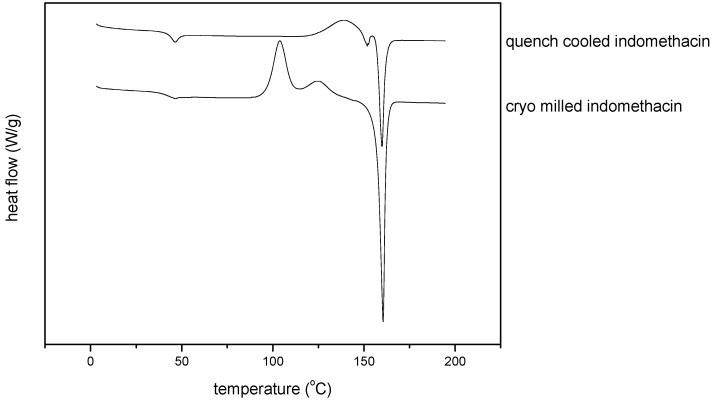
Thermograms of quench cooled amorphous and cryo milled amorphous indomethacin.

Thermodynamic differences in the amorphous state could be confirmed by DSC, as the thermograms of the quench cooled amorphous and cryo milled amorphous indomethacin were distinct from each other (Figure 5).

The glass transition temperature (T_g_) could be detected at 43.2 ^o^C (±1.1) for quench cooled indomethacin and at 39.7 ^o^C (±2.9) for cryo milled indomethacin, which was significantly lower (α = 0.05, P = 0.004; n = 3). No significant difference could be detected for the heat capacity change ∆c_p_ at the glass_transition though, with ∆c_p_ = 0.45 J/g^o^C (±0.06) for quench cooled indomethacin and ∆c_p_ = 0.49 J/g^o^C (±0.11) for cryo milled indomethacin (α = 0.05, P = 0.337; n = 3).

The thermograms of quench cooled and cryo milled indomethacin also showed differences in the exothermic recrystallisation events before melting and the endothermic melting event itself. Whereas quench cooled indomethacin showed only one exothermic event (the recrystallisation peak), cryo milled indomethacin showed first an exothermic event (at a lower recrystallisation temperature) immediately followed by another exothermic event before melting. The melting event itself also varies between the differently prepared amorphous forms; for quench cooled indomethacin two separate endothermic peaks occur, whereas for the cryo milled indomethacin only one peak could be observed. The thermograms are interpreted as follows: the cryo milled sample shows α and γ form recrystallising and then the γ form melting, whereas for the quench cooled amorphous indomethacin only the recrystallisation of the α form occurs, which consequently melts before recrystallising to the γ form and subsequent melting. However, it has to be taken into consideration that recrystallisation phenomenon for melt quenched compounds are cooling rate dependent [23].

In summary, this above data show that one should always bear in mind that the results for process induced disorder for a compound may be standard dependent. In the described experiments there is no absolute standard for calibration for the amorphous form. Furthermore milling does not necessarily result in amorphisation, but also intermediate disordered forms, hence the shortcoming of amorphous/ crystalline binary mixtures for calibration of process induced disorder in milled samples. Thus, quantification will always be standard dependent as the standard itself as well as the response of the analytical method to that standard will influence the calibration result and therefore the quantification outcome.

### 3.3. Influence of the analytical method on the quantification outcome for process induced disorder

Another multivariate analytical tool, PLS, can be used to quantify different solid state forms and has been applied for the same purpose in this study [24,25,26,27].

#### 3.3.1. PLS modeling of Raman spectroscopic data of quench cooled amorphous/ crystalline binary mixtures of indomethacin and simvastatin

**Figure 6:**
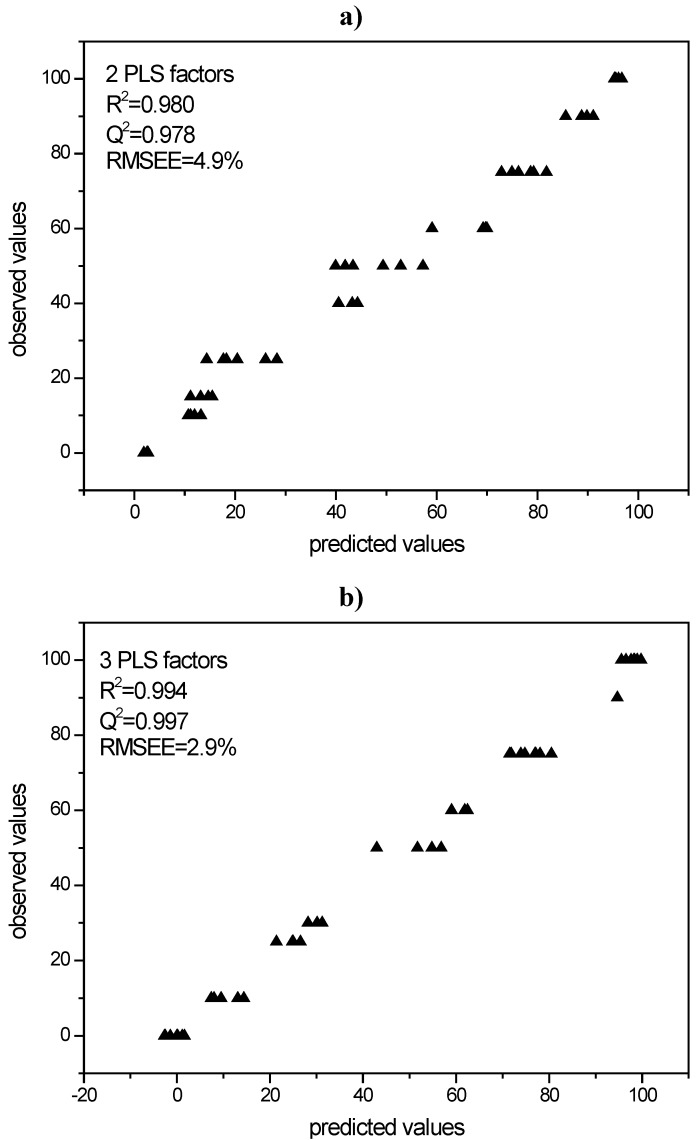
**a)** PLS model for Raman spectroscopic data of indomethacin according to cross validation; **b)** PLS model for Raman spectroscopic data of simvastatin according to cross validation.

PLS models were built in order to use them for the quantification of process induced disorder in the milled samples. For the indomethacin model two PLS factors were chosen with a resulting correlation coefficient R^2^ of 0.980 and a validation coefficient of Q^2^ = 0.978. The respective RMSEE was 4.9% (Figure 6a).

For simvastatin a model with three PLS factors was chosen. This model gave an R^2^ of 0.994 and Q^2 ^of 0.997 with the RMSEE being 2.9% (Figure 6b).

**Figure 7:**
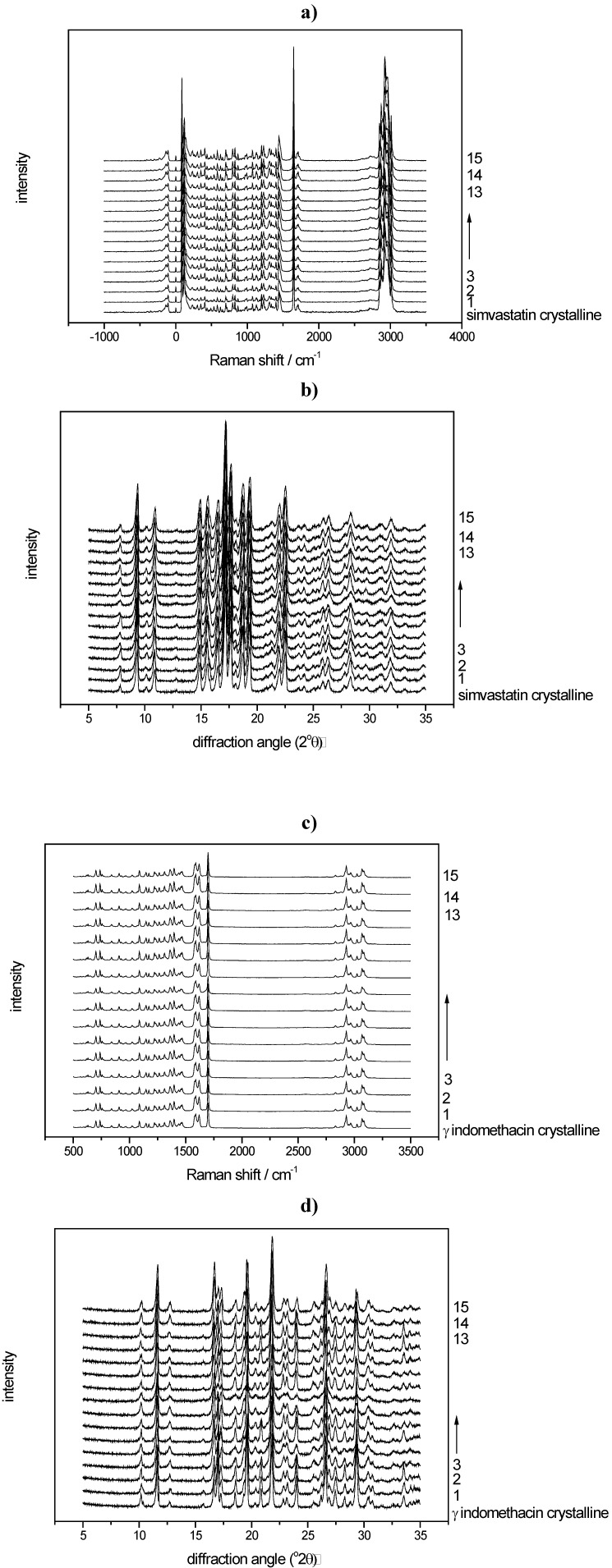
**a)** Raman spectra of the milled simvastatin samples and crystalline unprocessed simvastatin; **b)** Diffractograms of the milled simvastatin samples and crystalline unprocessed simvastatin; **c)** Raman spectra of the milled indomethacin samples and crystalline unprocessed indomethacin; **d)** Diffractograms of the milled indomethacin samples and crystalline unprocessed indomethacin.

All Raman spectra of the milled samples were analysed using the created PLS models. The sample processed under the harshest milling conditions (milled for 60 min with 60 balls at 25 Hz), e.g., resulted in 10.8% crystalline disorder for indomethacin and 15.5% crystalline disorder for simvastatin (Table 1). The respective raw data are presented in Figure 7a-d.

**Table 1:** Percentage of process induced disorder determined by Raman spectroscopy, XRPD and DSC for ball milled simvastatin and indomethacin (n.d.: no T_g_ detected). Experiment number 15 is the centre point experiment of the central composite face centered design and has been replicated (n = 3), giving a standard deviation (SD) of ±0.4 for the Raman spectroscopic data of simvastatin; SD ±2.9 (XRPD, simvastatin); SD ±0.1 (Raman spectroscopy, indomethacin) and SD ±0.7 (XRPD, indomethacin).

Sample No	Time Minutes	Frequency Hertz	No of Balls	Raman Simvastatin	XRPD Simvastatin	DSC Sim-vastatin	Raman Indo-methacin	XRPD Indo-methacin	DSC Indo-methacin
1	5	5	3	0.4	0.5	n.d.	1.8	-1.7	n.d.
2	60	5	3	0.4	0.2	n.d.	1.5	3.9	n.d.
3	5	25	3	2.2	-2.4	n.d.	1.6	8.1	n.d.
4	60	25	3	7.5	10.7	n.d.	2.8	21.6	n.d.
5	5	5	60	-0.4	1.4	n.d.	1.9	0.6	n.d.
6	60	5	60	0.2	0.7	n.d.	1.7	6.6	n.d.
7	5	25	60	3.7	8.0	n.d.	1.9	16.0	n.d.
8	60	25	60	15.5	18.3	n.d.	10.8	44.0	17.8
9	5	15	31	1.4	-1.1	n.d.	1.8	9.8	n.d.
10	60	15	31	3.2	2.1	n.d.	1.6	12.3	n.d.
11	32.5	5	31	0.2	-0.6	n.d.	1.8	4.5	n.d.
12	32.5	25	31	7.3	7.9	n.d.	5.0	24.2	n.d.
13	32.5	15	3	0.0	0.5	n.d.	1.7	5.7	n.d.
14	32.5	15	60	1.9	2.2	n.d.	2.4	11.9	n.d.
15	32.5	15	31	1.7	1.7	n.d.	1.8	17.6	n.d.

#### 3.3.2. PLS modeling of XRPD data of quench cooled amorphous/ crystalline binary mixtures of indomethacin and simvastatin

**Figure 8:**
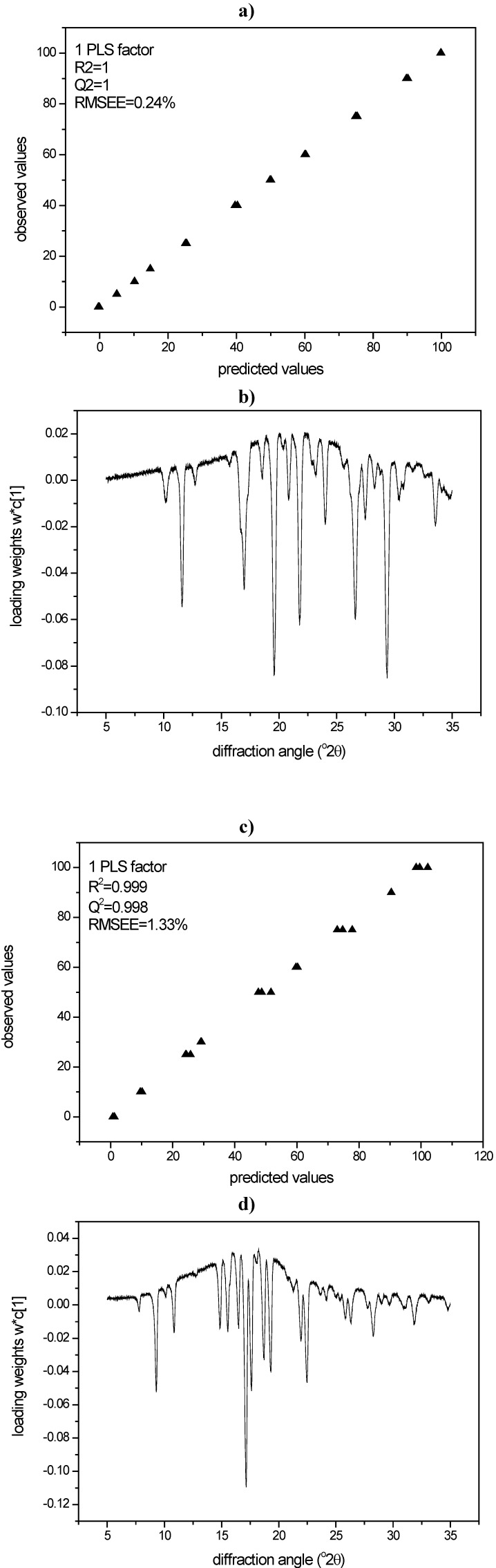
**a)** PLS model for XRPD data of indomethacin according to cross validation; **b)** Loading weights of the PLS factor (XRPD, indomethacin); **c)** PLS model for XRPD data of simvastatin according to cross validation; **d)** Loading weights of the PLS factor (XRPD, simvastatin).

PLS modeling of the diffractograms of the same binary mixtures was undertaken for the above mentioned quantification purpose. For the PLS models of both compounds, loadings and loading weights of the PLS factor were in good agreement and showed that in fact diffusive scatter (halo, positive loading values) as well as Bragg diffraction (peaks, negative loading values) were modeled (Figure 8a-d).

Consequently, whole XRPD diffractograms of the milled samples were analysed using the created PLS models. Using again the harshest milling conditions as an example (600 mg of compound milled for 60 min with sixty 4 mm balls at 25 Hz), the samples processed under these conditions showed 44.0% crystalline disorder for indomethacin and 18.3% crystalline disorder for simvastatin (Table 1). 

#### 3.3.3. DSC

**Figure 9:**
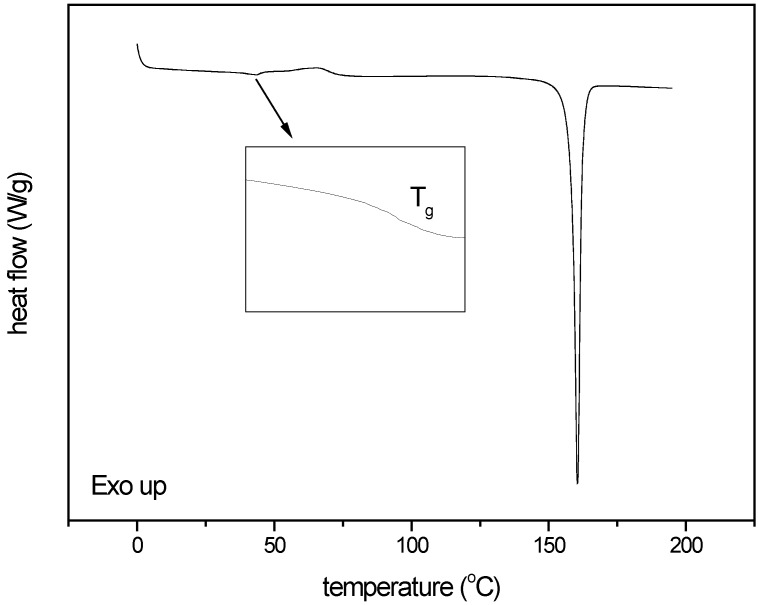
Thermogram of the indomethacin sample milled under the harshest milling conditions applied (milled for 60 min with 60 balls at 25 Hz).

For DSC analysis the change in heat capacity at the glass transition was used as a measure for amorphousness. The average change in heat capacity (∆c_p_) for the quench cooled, X-ray amorphous indomethacin samples was 0.45 J/g^o^C at the glass transition temperature (T_g_) of 43.2 ^o^C. Only in the indomethacin sample milled under the harshest milling conditions (600 mg of compound milled for 60 min with sixty 4 mm balls at 25 Hz) a glass transition, immediately followed by an exothermic event, *i.e.*, recrystallisation could be detected (Figure 9). The change in heat capacity for this sample was 0.08 J/g^o^C at T_g_ (42.4 ^o^C), corresponding to an amorphous content of 17.8%. 

For the quench cooled, X-ray amorphous simvastatin ∆c_p_ was 0.49 J/g^o^C (±0.01) at a T_g_ of 30.3 ^o^C (±0.9). For the entire set of milled simvastatin samples no glass transition could be detected, indicating that process induced changes in the ball milled samples did not lead to amorphisation. Only for the sample milled under the harshest milling conditions (milled for 60 min with 60 balls at 25 Hz) an exothermic recrystallisation event could be observed at 49.5 °C. Although no glass transition could be detected for the milled simvastatin samples, the one recrystallisation event in the sample with the harshest milling conditions can be interpreted as recrystallisation of a disordered form and therefore points to some kind of crystalline disorder. This finding was confirmed by Raman spectroscopy and XRPD (Table 1).

For other simvastatin samples as well as for indomethacin samples without a glass transition, process induced disorder was detected by Raman spectroscopy and XRPD. 

The comparison of the results for process induced disorder detected by Raman spectroscopy, XRPD and DSC revealed that the respective outcome regarding the content of process induced disorder in the ball milled drugs strongly depends on the analytical method used. Of all methods investigated, XRPD is the one that tends to give a lower percentage of crystallinity than Raman spectroscopy and DSC. 

We understand these discrepancies as being method inherent. Thermal analysis detects the existence of the truly amorphous state, defined as a second order phase transition in form of the glass transition. In contrast, XRPD predominately detects crystallinity, the absence of which may be due to several forms of process induced disorder (“total process induced disorder”). Also, the decrease of crystalline peaks may be due to particle size effects, as very small particles may lead to weaker diffraction peaks and bigger particles may facilitate preferred orientation, which again may influence peak intensities [8]. Thus, PLS modeling of XRPD data in order to quantify the amount of process induced disorder could be problematic as the method might not differentiate between changes in the diffractograms induced by crystalline disorder and diffractogram changes that are induced by changes in particle size. Consequently, quantification of the solid state of milled drugs by XRPD may be biased.

### 3.4. Influence of the drug itself on the quantification outcome for process induced disorder

For simvastatin the differences between crystalline disorder detected by Raman spectroscopy and XRPD were not as distinct as for indomethacin. This phenomenon could be attributed to the drugs itselves. Quench cooled amorphous indomethacin exists predominantly as dimer as does the γ crystalline form [28]. Hydrogen bonding in simvastatin facilitates the formation of molecule chains along the *a* axis in the crystal [29]. The remaining near order of quench cooled simvastatin has not been published to the best of our knowledge, but it is suspected that the differences between crystalline and amorphous near order are more distinct for simvastatin than for indomethacin. This could explain the more consistent Raman and XRPD results for simvastatin, as Raman spectroscopy being a near order analytical method might not easily pick up on disordered states, where the near order of amorphous and crystalline form are very similar.

Taken together, our findings suggest the presence of different disordered states in the milling process. On the basis of the different results obtained by the different analytical methods, existence of pre-amorphous (without exhibition of a glass transition) and amorphous (with glass transition) disordered states are assumed. 

## 4. Conclusions

This study has compared three different techniques, namely XRPD, DSC and Raman spectroscopy, with respect to the quantification of the content of processed induced crystalline disorder in a milling process. Care must be taken when interpreting the results obtained with these three methods. Within XRPD the assignment of changes in the diffractogram due to “total process induced disorder” (*i.e.*, amorphous content and other forms of crystalline disorder) and particle size effects appears to be problematic. However, XRPD is the method of choice for the detection of remaining crystallinity. For the detection of the truly amorphous state, defined by a glass transition, DSC is the method of choice. Raman spectroscopy combined with multivariate analysis detects and quantifies crystalline disorder before the compound can be described as DSC amorphous as well as thereafter. However, Raman spectroscopy as a molecular level technique is sensitive to the near order of solid materials and therefore could ‘underestimate’ the degree of disorder if the material remains near range ordered to a certain extent (*i.e.*, exists as a dimer) in the amorphous state. Thus, for the quantification purpose of milled drugs Raman spectroscopy combined with multivariate analysis appears to be advantageous for drugs with significant differences in the near order of the crystalline and amorphous form as particle size effects are not as apparent and the whole range of crystalline disorder is covered. However, to understand the investigated system fully, all methods need to be used complementary.
